# Quantitative assessment of radiation dose and fractionation effects on normal tissue by utilizing a novel lung fibrosis index model

**DOI:** 10.1186/s13014-017-0912-y

**Published:** 2017-11-07

**Authors:** Cheng Zhou, Bleddyn Jones, Mahmoud Moustafa, Christian Schwager, Julia Bauer, Bing Yang, Liji Cao, Min Jia, Andrea Mairani, Ming Chen, Longhua Chen, Juergen Debus, Amir Abdollahi

**Affiliations:** 1 German Cancer Consortium (DKTK), Translational Radiation Oncology, National Center for Tumor Diseases (NCT) and German Cancer Research Center (DKFZ), INF 460, 69120 Heidelberg, Germany; 2 Department of Radiation Oncology, Heidelberg Ion-Beam Therapy Centre (HIT), University of Heidelberg Medical School, Heidelberg, Germany; 3 Heidelberg Institute of Radiation Oncology (HIRO), National Center for Radiation research in Oncology (NCRO), Heidelberg, Germany; 4 Department of Radiation Oncology, Nanfang Hospital, Southern Medical University, Guangzhou, China; 5 Gray Laboratory, CRUK/MRC Oxford Oncology Institute, Radiation Oncology, University of Oxford, Oxford, UK; 6 Department of Clinical Pathology, Suez Canal University, Ismailia, Egypt; 7 Physics Institute University of Heidelberg, Heidelberg, Germany; 8 Inviscan SAS, Strasbourg, France; 9 Division of Clinical Epidemiology and Aging Research, German Cancer Research Center (DKFZ), Heidelberg, Germany; 10 Zhejiang Key Lab of Radiation Oncology, Zhejiang Cancer Hospital, Hangzhou, China; 11 Italian National Center for Oncological Hadron Therapy (CNAO), Pavia, Italy

**Keywords:** Thoracic radiotherapy, Lung fibrosis, Fractionation, BED, α/β ratio

## Abstract

**Background:**

Normal lung tissue tolerance constitutes a limiting factor in delivering the required dose of radiotherapy to cure thoracic and chest wall malignancies. Radiation-induced lung fibrosis (RILF) is considered a critical determinant for late normal tissue complications. While RILF mouse models are frequently approached e.g., as a single high dose thoracic irradiation to investigate lung fibrosis and candidate modulators, a systematic radiobiological characterization of RILF mouse model is urgently needed to compare relative biological effectiveness (RBE) of particle irradiation with protons, helium-, carbon and oxygen ions now available at HIT. We aimed to study the dose-response relationship and fractionation effect of photon irradiation in development of pulmonary fibrosis in C57BL/6 mouse.

**Methods:**

Lung fibrosis was evaluated 24 weeks after single and fractionated whole thoracic irradiation by quantitative assessment of lung alterations using CT. The fibrosis index (*FI*) was determined based on 3D-segmentation of the lungs considering the two key fibrosis parameters affected by ionizing radiation i.e., a dose/fractionation dependent reduction of the total lung volume and increase of the mean lung density.

**Results:**

The effective dose required to induce 50% of the maximal possible fibrosis (*ED*_*50*_) was 14.55 ± 0.34Gy and 27.7 ± 1.22Gy, for single and five- fractions irradiation, respectively. Applying a deterministic model an α/β = 4.49 ± 0.38 Gy for the late lung radiosensitivity was determined. Intriguingly, we found that a linear-quadratic model could be applied to in-vivo log transformed fibrosis (*FI)* vs. irradiation doses. The LQ model revealed an α/β for lung radiosensitivity of 4.4879 Gy for single fraction and 3.9474 for 5-fractions. Our *FI* based data were in good agreement with a meta-analysis of previous lung radiosensitivity data derived from different clinical endpoints and various mouse strains. The effect of fractionation on RILF development was further estimated by the biologically effective dose (BED) model with threshold BED (*BED*_*Tr*_) = 30.33 Gy and *BED*_*ED50*_ = 61.63 Gy, respectively.

**Conclusion:**

The systematic radiobiological characterization of RILF in the C57BL/6 mouse reported in this study marks an important step towards precise estimation of dose-response for development of lung fibrosis. These radiobiological parameters combined with a large repertoire of genetically engineered C57BL/6 mouse models, build a solid foundation for further biologically individualized risk assessment of RILF and functional RBE prediction on novel of particle qualities.

**Electronic supplementary material:**

The online version of this article (10.1186/s13014-017-0912-y) contains supplementary material, which is available to authorized users.

## Background

Radiotherapy is an integral component for treatment of thoracic tumors and breast cancer, however, the high sensitivity of normal lung tissue to ionizing radiation (IR) leading to long term sequela such as development of pulmonary fibrosis constitutes a major dose limiting constraint for a curative treatment [1]. More recently, hypofractionated stereotactic body or ablative radiation therapy (SBRT, SABR) is increasingly used for e.g., early stage non-small cell lung cancer (NSCLC) and oligometastatic diseases [2, 3]. SBRT is characterized by intensified deposition of radiation doses into one or few circumscribed regions in a single or few fractions [4]. In NSCLC e.g., a biologically effective dose (BED) of ≥ 100Gy is aimed in 1-8 fractions (based on tumor localization) with excellent local control rates [5, 6]. The high focused dose is delivered at the expense of a relatively high prescribed dose to the normal lung tissue. Therefore, several approaches are undertaken to estimate the region at risk for long term lung tissue complications after SABR/SBRT [7, 8]. To minimize normal tissue toxicity, dose volume histogram (DVH) based evaluation are considered, e.g., mean lung dose (MLD ≤ 20Gy) and total lung V20 dose-volume constraints being ≤35% according to the recommendation of National Comprehensive Cancer Network (*NCCN v4.2016*). These dose-volume constraints are mostly empirically based and lack of rigorous preclinical validation. Therefore, there is an urgent need for a systematic characterization and radiobiological modeling of radiation induced pulmonary fibrosis in experimental- and clinical settings for a better understanding and estimation of lung tolerance to ionizing radiation.

The clinical sequelae of radiation injury consist of a subacute onset of radiation-induced inflammation (pneumonitis) with later activation of the fibrogenesis processes [9, 10]. Preclinical studies of radiation-induced lung toxicity in different mouse models [11–15] has improved our understanding of the pathophysiology of radiation lung toxicity and led to the development of biophysical models [16–22]. Among those classical studies, two physiological parameters were most frequently applied for surrogating lung damages induced by ionizing radiation, breathing rate (*breaths per minute, BPM*) and the lethality (*LD*_*50*_). By integrating computer assisted radiology, the present study utilized a novel CT imaging based surrogate, fibrosis index (*FI*) algorithm for the quantitative assessment of lung fibrosis. C57BL/6 mice is among the most frequently studied experimental models of lung fibrosis [23], however, the key parameters determining the lung tissue radiosensitivity were missing. Our data now provide radiobiological estimates for α/β ratio and BEDs for the endpoint of radiation induced pulmonary fibrosis in this important preclinical model.

This work was conducted in frame of German Research Foundation (DFG) “clinical research group heavy ion therapy (KFO-214)” in collaboration between the project TP5 and the central platform (ZP1). It builds the conceptual basis for accurate estimation of relative biological effectiveness (RBE) for carbon ions. Data on RBE variation as a function of fractionation and linear energy transfer (LET) utilizing the here presented frame work are in preparation for publication.

## Methods

### Irradiation and animals

Whole thoracic irradiation was administrated to female C57BL/6 mice (Charles River Breeding Laboratories, MA) aged between 8 and 10 weeks. All animal work was approved and performed in compliance with rules outlined by the local and governmental animal care committee instituted by the German government (Regierungspraesidium, Karlsruhe). Photon irradiation was delivered by a 6 MeV Artist Linac (Siemens, Germany) at a dose rate of 3 Gy/min. Prior to thoracic irradiation, mice were anaesthetized by an intraperitoneal application of 0.36 ml/kg Rompun 2% (Bayer HealthCare) and 0.54 ml/kg ketamine 10% (Pfizer). Ten anesthetized mice were placed in a specially constructed Polymethylmethacrylat (PMMA) holder for immobilization and irradiated simultaneously. To ensure full coverage of the lung field with breathing motion and sparing neighboring tissues at the maximum, the irradiation plans were adjusted by anatomical and radiological measurement. Dosimetry was used to confirm the dose uniformity in advance.

### Experimental design

A wide range of dose series were included in the dose-escalation trials, single fractions (1-fx) arm of: 0, 10.5, 12.5, 14.5, 17.5, 20 Gy; five fractions (5-fx) arm of: 0, 2, 4, 6, 7, 8.5 Gy per fraction. Fractions were given once a day. Each dose group contained 12 randomly grouped mice. Quantitative CT imaging was performed every 4 weeks post irradiation. Based on our previous experiments week 24 was revealed as a suitable interval after radiation to determine late fibrosis development in terms of radiological, histological findings and lethality [1, 24–26]. Mice with signs of severe dermatitis were sacrificed according to the ethics of local governmental animal care committee.

### Assessment of lung fibrosis by computed tomography (CT)

A clinical PET/CT scanner (Biograph mCT, Siemens) was applied for quantitative CT imaging pre- and post-irradiation. The standard protocol employed for the CT portion of PET/CT was as follows: 80 kV with 80 mAs, a pitch of 0.6 mm, slice thickness of 0.6 mm and acquisition time of 32 s. X-ray exposure is approximately 4.14 mGy per scan. Images were reconstructed using the filter kernel H50s into a transaxial FOV of 138 × 138 mm^2^ as a 512 × 512 matrix. Images acquired from the clinical CT scanner were viewed and analyzed in MITK software. The lung tissue density was measured by average Hounsfield unit (HU) intensities. The lung, together with all the micro-structures, was thereby segmented using a 3D regional growing algorithm with a lower threshold of −900 HU and an upper threshold of −100 HU. Trachea and primary bronchi were manually resected upon segmentation. Volume sizes and mean HU values within the segmented area were calculated for quantitative assessment of pulmonary toxicity. The fibrosis index was employed to assess the extent of fibrosis as the major endpoint. Briefly, the *FI* model is based on two critical parameters derived from CT segmented data: the relative increase in mean lung density (*∆HU*) and decreased lung volume (*∆V*) when compared to the mean of an age-matched reference mice cohort. Biologically, the augmented *ΔHU* is an overall representation of collagen deposition and increased cellularity; whereas *∆V* reflects the nature of fibrosis as a restrictive lung disease. The calculation of *FI* is based on the proposed equation as:

1 $$ \mathrm{Fibrosisindex}\left(\mathrm{FI}\right)=\sqrt{\varDelta \overline{\mathrm{HU}\uparrow}\times \varDelta \overline{\mathrm{V}\downarrow }} $$

**Fig. 1:**
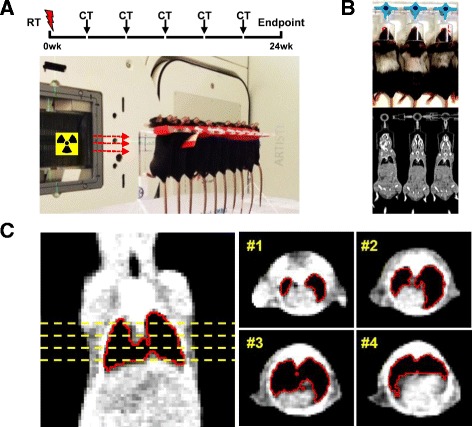
Overview of the experimental setup. **a** Simultaneous whole thoracic irradiation with 6 MV Linac (red arrows, anterior-posterior direction of horizontal beam) of up to 10 mice fixed with stretched thoraces in vertical position on a custom holder. Radiotherapy (RT) was followed by longitudinal CT imaging every 4 weeks over an overall period of 24 weeks (Endpoint). **b** CT-scan of animals under inhalation anesthesia. Note the thoracic area with gray hairs, lack of melanin, indicating the irradiated region. **c** Processing of CT-images; the lung area was semi-automatically segmented (red line) and manually curated slice by slice. Total lung volume (mm^3^) and mean lung density (HU) provided the basis for calculation of the lung fibrosis index (FI) as indicated in the methods section. (FI = fibrosis index, HU = Hounsfield unit, RT = radiotherapy, fx = fractionation)

The presence of radiation fibrosis at 24 weeks post irradiation was determined at the endpoint using delta HU and delta V via the segmentation of the entire lung (Fig. 1). Caution is warranted in the case of combined pulmonary fibrosis and emphysema syndrome (CPFE) or pleural effusions. The mean lung density as well as lung volume based on CT measurements might be biased due to the presence of emphysema (air) or effusions (fluids). A supplemented CT histograms analysis, ‘peak position of smoothened histogram (PPSH)’ was used for a differential diagnosis (see Additional file 1: Appendix).

### Analysis of data

Single and fractionated *FI*s data were fitted by a modified probit model derived from Kallman et al. [27] using OriginPro 8.0 and Mathematica Software 9.0.

2 $$ \mathrm{FI}\left(\mathrm{D}\right)=\frac{1}{2}\mathrm{A}\left\{1-\operatorname{erf}\left(\sqrt{\uppi}\upgamma\ \left(1-\frac{\mathrm{D}}{{\mathrm{ED}}_{50}}\right)\right)\right\} $$

where *A* is the saturation constant for maximal development of fibrosis measured experimentally to be 7.20 (equal to 100% fibrosis), serving to quantize all *FI*s. Of note, the *FIs* versus dose data set used here was continuous rather than event data, hence a deterministic model was applied. Therefore, unlike *ED*_*50*_ applied in probabilistic models corresponding to the probability for half (50%) of the population (animals) to develop an event, the fibrosis *ED*_*50*_ could be here interpretated as the dose where the whole population experiences an average 50% increase of the *FI* (*FI* = 3.60) relative to maximum possible effect (*FI* = 7.20). *γ* is the maximum value of the normalized dose-response gradient. By integrating *FI*s into the late lung toxicity analysis, eq. (2) was proposed as *FI-model* for short.

The parameter for fractionation sensitivity α/β ratio was derived from the concept of biologically effective dose (BED) [28]. Equivalent BEDs can be achieved by different isoeffective fractionation regimens as:

3 $$ {\mathrm{D}}_1\left(1+\frac{{\mathrm{d}}_1}{\upalpha /\upbeta}\right)={\mathrm{D}}_2\left(1+\frac{{\mathrm{d}}_2}{\upalpha /\upbeta}\right) $$

Solving eq. (3), we get α/β as:

4 $$ \frac{\upalpha}{\upbeta}=\frac{{\mathrm{D}}_2{\mathrm{d}}_2-{\mathrm{D}}_1{\mathrm{d}}_1}{{\mathrm{D}}_1-{\mathrm{D}}_2} $$

Hence, the value of α/β can be obtained by two paired dose values, *D*_*1*_ and *D*_*2*_ giving rise to the same biological effect (namely the equal *FI*). According to the *FI*-model, any *D*_*X*_ can be determined by the inverse function of eq. (3):

5 $$ {\mathrm{D}}^{-1}(FI)={ED}_{50}\left[1-\frac{1}{\sqrt{\uppi}\upgamma}{\operatorname{erf}}^{-1}\left(1-\frac{2 FI}{\mathrm{A}}\right)\right] $$

As a result, for any given *FI* value (0.05 ≤ *FI* ≤ 7.20), we can derive corresponding *D*_*1*_ and *D*_*2*_ values with reference to single and five fractionation schedules. The estimation of α/β was eventually made by referring to eq. (4) within the effective range of *FI*s.

The *FI*s data was also analysed as a function of BED doses by logistic regression as:

6 $$ \mathrm{FI}\left(\mathrm{BED}\right)=\mathrm{A}2+\frac{\left(A1-A2\right)}{\left[1+\left({\left( BED/k\right)}^p\right)\right]} $$

The threshold of BED to initiating fibrosis (*BED*_*Tr*_) was defined mathematically as the maximum curvature of the curve; whereas the cut-off dose was derived from the maximum slope.

The simulated data of α/β ratios as well as iso-effect doses were fitted with an exponential decay function. For a determination of radiobiological parameters (i.e., α, β, α/β), the *FI* data were Log transformed and fitted to the linear-quadratic (LQ) model [18].

### Literature review and statistical analysis

Literature studies were reviewed with reference to radiobiological modeling of late lung damage in mouse models. A total of 13 articles were included and the detailed parameters (i.e. author, publication year, animal, α/β ratio, endpoint, follow-up time) were extracted. Forest plots were applied to interpret the values of α/β ratio in all studies (*R software v1.5.1*). Data is presented as mean ± SD or otherwise stated. *P* < 0.05 is considered as statistically significant.

## Results

### Dose-response curves and fibrosis *ED*_*50*_

**Fig. 2:**
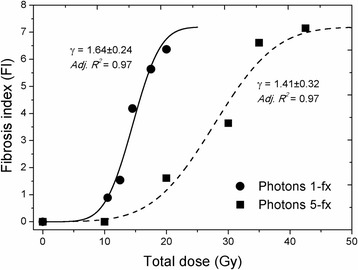
Dose-response modeling of pulmonary fibrosis induction after single and five fractions of photon irradiations. The sigmoidal curves were plotted based on fibrosis index (FI) (*Adjusted R* ^*2*^=0.972, 0.968 for 1-fx and 5-fx curve, respectively). Single fraction photon doses are shown as solid circles and five fractionated as solid squares

The dose-response curves of RILF using the *FI-model* is demonstrated (Fig. 2). The radiation effect curve of single dose was much steeper compared to fractionated irradiation (*γ = 1.64 ± 0.24, 1.41 ± 0.32, respectively; Adj. R* ^*2*^ *=0.97, 0.97, respectively*). Fibrosis development, as surrogated by *FI* was markedly enhanced above a threshold dose of 11 Gy. In contrast, normal lung tissue was better spared from radiation injury using fractionated schedules (5-fx). The fibrosis *ED*_*50*_ (effective dose for 50% fibrosis or *FI* = 3.60) for single- and five- fractions irradiations were identified to be 14.55 ± 0.34 Gy and 27.7 ± 1.22 Gy, respectively. This indicates an elevated tolerance of normal lung tissue to fractionated photons exposure, in that increasing physical doses were required for the same effectiveness.

### α/β, isoeffect curves and threshold BED

**Fig. 3:**
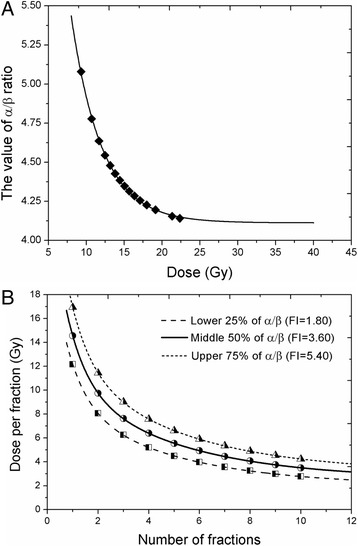
Simulation based estimation of alpha/beta ratio. Estimates of radiobiological parameters according to simulated iso-effect data revealed that the value of α/β ratio for normal lung tissues varies with irradiated doses. **a** Dose per fraction is plotted against the number of fractions (**b**). Isoeffect curves with reference to the median, first and third quartiles of α/β ratios are shown

The fractionation sensitivity related parameter, the α/β ratio of lung was estimated using biologically effective dose (BED) equations [28]. The obtained value of α/β ratio appears to vary with doses, and was found to be 4.49 ± 0.38 Gy based on *FI-model* (Fig. 3a). The α/β ratio gradually approaches to 4.26 Gy (at fibrosis *ED*_*75*_) and even 4.20 Gy (at fibrosis *ED*_*90*_) at severe fibrosis level. According to this analysis, using simulated data by the “direct quantal” method [19], the α/β ratio obtained at fibrosis *ED*_*50*_ was estimated to be 4.38 Gy (Additional file 1: Figure S1). The iso-effect dose curve with reference to the median, first and third quartiles of the α/β ratios are provided (Fig. 3b).

**Fig. 4:**
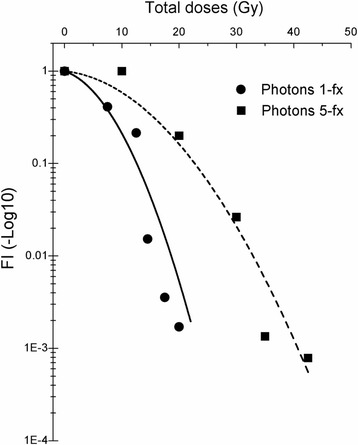
Discovery of a linear quadratic response of lung tissue to whole thoracic irradiation. The LQ-model was applied to fit the in-vivo lung tolerance data, i.e., the degree of fibrosis development determined by *FI*s after single* vs.* fractionated irradiation. The negative log10 transformed *FI* values are plotted as a function of the total prescribed doses. The LQ parameters were derived as: α = 0.048 Gy^−1^, β = 0.010 Gy^−2^, α/β = 4.800 Gy for single fraction; α = 0.015 Gy^−1^, β = 0.0038 Gy^−2^, α/β = 3.947 Gy for five fractions. Single fraction photon doses are shown as solid circles and 5 fractionated as solid squares

On the other hand, by fitting to LQ model, the principle radiobiological parameters, α/β, α, β were also obtained approximately as α/β = 4.4879 Gy, α = 0.0480 Gy^−1^ and β = 0.0107 Gy^−2^ for single dose and α/β = 3.9474 Gy, α = 0.0150 Gy^−1^ and β = 0.0038 Gy^−2^ for 5 fractionated irradiation (Fig. 4).

**Fig. 5:**
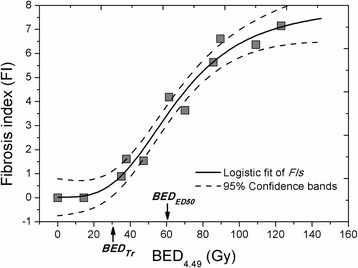
Biologically effective dose (BED) based dose-response modeling of radiation-induced lung fibrosis. The fibrosis index is plotted against the biological effective doses (BEDs) (*Adj. R* ^*2*^=0.959, 95% confidence interval was given within dot lines). Threshold BED (*BED*_*Tr*_) as well as BED for developing 50% of fibrosis (*BED*_*ED50*_) is indicated by the arrows

BEDs with respect to all irradiated doses were predicted based on the derived α/β of 4.49 Gy. The dose-response relationship between fibrosis development and BEDs is shown (Fig. 5). The threshold BED (*BED*_*Tr*_) to trigger or initiate lung fibrosis was identified as 30.33 Gy. The cut-off BED dose was 54.23 Gy. The *BED*_*ED50*_ (BED results in 50% of fibrosis) was determined as 61.63 Gy.

### Systematic review of lung α/β ratios

**Fig. 6:**
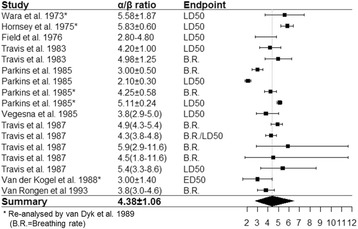
A systematic review and meta-analysis of α/β ratios from previous mouse experiments considering different surrogates for late radiation induced lung damage. An average of 4.38 ± 1.06 Gy was estimated from this pooled analysis. The detailed characteristics of included literatures are provided in Additional file 1: Table S1. Data are presented as Mean ± SE

A meta-analysis of experimentally derived lung α/β ratios from published studies is shown as a forest plot (Fig. 6). Detailed characteristics of the 13 studies included are provided (Additional file 1: Table S1) [15, 16, 21, 22, 29–37]. The value of α/β ratio may vary with different functional assays, endpoints, follow-up time and biophysical models. The α/β ratio discovered by the *FI*-model in our study was consistent with the estimated α/β of 4.38 ± 1.06 derived from this pooled analysis. Given that different physiological parameters were utilized in the reports included in the meta-analysis, e.g., breath rate and *LD*_*50*_, the high agreement of the α/β values underscores the robustness of our CT based *FI-model* to assess lung radiosensitivity.

## Discussion

The high sensitivity of lung tissue to ionizing radiation induced damage constitutes a major obstacle for curative radiotherapy of thoracic tumor. Therefore, a better characterization of radiation induced late effects in lung tissue is of utmost interest for the field of radiotherapy. Lung is featured as a late-responding tissue. In accordance with clinical observations, our data suggest a great sensitivity of late responses to changes in fractional dose. Late toxicity was significantly attenuated after 5-fractionated doses and the determined fibrosis index changes could be fitted by the LQ model. Preclinical data are limited in evaluating the late lung toxicity occurred with the intent of deriving isoeffective doses. The isoeffect curves presented here imply that the size of dose per fraction (or the fraction number) plays an essential role in sparing late lung injury. Taken together, our data clearly indicated an unfavourable toxicity profile for delivering a large fractionated dose to normal lung, unless the total dose is carefully selected.

Quantitative estimates of radiobiological characteristics for late phase of pulmonary fibrosis are urgently needed for a better experimental design of translational research in this area. A rough estimate of murine lung α/β ranging from 2.4-6.3 Gy was given by Fowler [38]. The α/β determined in the present study is 4.49 ± 0.38 Gy; While at the dose of fibrosis *ED*_*50*_, the α/β was estimated ~ 4.38 Gy. Intriguingly, pooled analysis from the past literature indicates a consensus of α/β at 4.38 ± 1.06 Gy. Furthermore, the precise dose-response relationship between BED and fibrosis development in mouse was firstly illustrated in this paper. The high comparability of the here presented late lung toxicity data with current empirically derived clinical BED data suggest further exploration of this model including other known key modulators of pulmonary sensitivity to ionizing irradiation such as combination regimens (e.g. radiochemotherapy) or partial volume effects.

Knowledge of the precise value of the average human lung α/β ratio is clinically essential, but there is no clear consensus. As reviewed by Bentzen et al., a wide range of α/β ratios from 0.9 to 8.5 Gy was reported by different clinical studies based on conventional radiotherapy [39]. In the setting of SBRT, an α/β ratio of 3 Gy is most frequently used [40]. Discrepancies between the ~4.4 α/β ratio obtained under experimentally controlled mouse condition *vs.* different human data may be explained by variability of numerous parameters, i.e., variable lung volumes and positions, cardiac exposure, different surrogates (e.g., pneumonitis or radiographic changes) and species specific responses. It is also possible that the α/β ratios could vary with age, cigarette smoking and other air pollution histories.

Among potential limitations of this study could be the restriction of quantitative CT-scan parameters such as lung density to discriminate between lung fibrosis *vs.* inflammation, lung damage induced secondary to cardiac dose, or breathing motions. However, these CT-derived parameters were found to correlate well with a broad spectrum of histopathological and molecular surrogates of lung fibrosis [1, 24, 26]. Further, lethality as a function of irradiation doses was not investigated in this study. Prediction of α/β ratio based on LQ model requires a more solid mechanistic basis, as this model so far links the radiation dose with cell survival and repopulation effects.

With advance of particle beam irradiation, the biological evaluation of (sub-) cellular and tissue response to photons, protons and carbon-ions irradiation is urgently needed [41–44]. However, preclinical in-vivo comparison of normal tissue effects such as RILF as a function of different radiation qualities are missing. Hence, the proposed *FI-model* builds a solid bio-math-physical foundation for experimental RBE modeling.

## Conclusion

In conclusion, we have introduced CT imaging based *FI-model*, providing a quantitative description of radiobiological characteristics as well as the dose-sparing effect of fractionation in a murine model. The α/β ratio for fibrosis induction was extracted, in parallel with a pooled analysis from a literature review. Significant sparing of late lung toxicity was illustrated in a fractionated dose regime and threshold “tolerance BED dose” was also determined. Given the spectrum of genetic mouse models available in the here employed C57BL/6 background, our data will impact design and development of personalized normal tissue toxicity estimation and targeted therapeutic interventions. The here reported radiobiological characterization of this model further provides a starting point for determining the RBE for RILF of novel raster scanning proton, helium, carbon and oxygen ions available at HIT.

## Additional files

Additional file 1: **Table S1.** A list of experimentally derived mouse lung α/β ratios from the literatures with special reference to late lung damage. Data is presented as Mean ± SE. (*B.R. =* breathing rate, *F* = female, *BPM* = breath per minute, *M* = male, *d* = day, *wk.* = week). **Figure S1.** Reciprocal total isoeffect dose for *ED*_*50*_ as a function of dose per fraction. The data points were simulated using eq. (3). The α/β was obtained as the ratio of the intercept and the slope of the line using the conventional Fe plot.** Appendix.** CT histogram profiling in differential diagnosis of emphysema or pleural effusions. (DOCX 350 kb)

## Abbreviations

BED: biologically effective dose
BPM: breaths per minute
CT: computed tomography
DVH: dose volume histogram
FI: fibrosis index
HU: Hounsfield unit
LET: linear energy transfer
LQ: linear quadratic model
MLD: mean lung dose
NSCLC: non-small cell lung cancer
PMMA: Polymethylmethacrylat
RBE: relative biological effectiveness
RILF: Radiation-induced lung fibrosis
SBRT or SABR: hypofractionated stereotactic body or ablative radiation therapy
V20: % total lung volume receiving ≥20Gy
α/β: alpha/beta ratio
