# Supplementary material for: Quantitative assessment of radiation dose and fractionation effects on normal tissue by utilizing a novel lung fibrosis index model
Source: Radiat Oncol. 2017 Nov 7;12:172. doi: 10.1186/s13014-017-0912-y (PMC5678815; doi:10.1186/s13014-017-0912-y)
Supplement: Supplementary file 1 — Table S1. A list of experimentally derived mouse lung α/β ratios from the literatures with special reference to late lung damage. Data is presented as Mean ± SE. (B.R. = breathing rate, F = female, BPM = breath per minute, M = male, d = day, wk. = week). Figure S1. Reciprocal total isoeffect dose for ED 50 as a function of dose per fraction. The data points were simulated using eq. (3). The α/β was obtained as the ratio of the intercept and the slope of the line using the conventional Fe plot. Appendix. CT histogram profiling in differential diagnosis of emphysema or pleural effusions. (DOCX 350 kb) [file 13014_2017_912_MOESM1_ESM.docx]

**Index**

- Supplementary Table S1
- Supplementary Figure S1
- Appendix

# Supplementary Table S1

| **Animals** | **α/β ratio (Gy)** | **Endpoint** | **Follow-up** | **Reference** |
| --- | --- | --- | --- | --- |
| LAF1 mouse (F) | 5.58±1.87* | LD_50_ | 100-160d (~23wk) | Wara *et al.* 1973 [16] |
| TO mouse (F) | 5.83±0.60* | LD_50_ | 60-200d (~28wk) | Hornsey *et al.* 1975 [29] |
| TO mouse (F) | 2.8-4.8 | LD_50_ | 180d (~24wk) | Field *et al.* 1976 [21] |
| CBA/Ht Gyf BSVS mouse (M) | 4.2±1.0 | LD_50_ | 28wk | Travis *et al.* 1983 [30] |
| CBA/Ht Gyf BSVS mouse (M) | 4.98±1.25 | B.R. (420 BPM)^¶^ | 28wk | Travis *et al.* 1983 [30] |
| CBA/Ht Gyf BSVS mouse | 3.0±0.5 | B.R. (×1.2) | 48wk | Parkins *et al.* 1985 [15, 31] |
| CBA/Ht Gyf BSVS mouse | 2.1±0.3 | LD_50_ | 48wk | Parkins *et al.* 1985 [15, 31] |
| CBA/Ht Gyf BSVS mouse | 4.25±0.58* | B.R. (×1.2) | 48wk | Parkins *et al.* 1985 [15, 31, 22] |
| CBA/Ht Gyf BSVS mouse | 5.11±0.24* | LD_50_ | 48wk | Parkins *et al.* 1985 [15, 31, 22] |
| C3Hf/Sed mouse | 3.8 (2.9, 5.0) | LD_50_ | 25wk | Vegesna *et al.* 1985 [32] |
| C3Hf/Kam mouse (M) | 4.9 (4.3, 5.4) | Pneumonitis (B.R.) | 80-224d (~32wk) | Travis *et al.* 1987 [33] |
| C3Hf/Kam mouse (M) | 4.3 (3.8, 4.8) | Lung damage (B.R., LD_50_) | 80-365d (~52wk) | Travis *et al.* 1987 [33] |
| LAF1/J mouse | 5.9 (2.9, 11.6) | B.R. (BPM≥12%) | 28wk | Travis *et al.* 1987 [34] |
| LAF1/J mouse | 4.5 (1.8, 11.6) | B.R. (BPM≥12%) | 48wk | Travis *et al.* 1987 [34] |
| LAF1/J mouse | 5.4 (3.3, 8.6) | LD_50_ | 48wk | Travis *et al.* 1987 [34] |
| CD-1 mouse (F) | 3.0±1.4* | Lethal pneumonitis (ED_50_) | 180d (~24wk) | Van der Kogel *et al.* 1988 [35] |
| C3Hf/Kam mouse (M) | 3.8 (3.0, 4.6) | Pneumonitis (B.R.^¶^) | 38wk | Van Rongen *et al.* 1993 [36] |

^¶^ B.R.=Breathing rate, BPM=breath per minute

* Re-analysed by van Dyk *et al.* [22, 37]

# Supplementary Figure S1





# Appendix

CT histogram profiling in differential diagnosis of emphysema or pleural effusions

The MLD and LV have to be used with caution in cases of combined pulmonary fibrosis and emphysema syndrome (CPFE) [1] or pleural effusions [2] . A histogram analysis of fibrosis development in lung over 24 weeks based on clinical CT measurement results was performed. From 12 weeks after photons irradiation, a remarkable right-shift of peak position of smoothened histogram (PPSH) value was found, implying a gradual increase of lung density over the following 12 weeks. PPSH defined technically here is as: after mean HU value and volume size were calculated within the segmented area, a histogram of the same lung region binned in an interval of 10 HU was extracted in order to achieve a more reliable evaluation which was insensitive to the selection of threshold values. This histogram was further smoothened using a negative exponential smoother with a sampling proportion of 0.2. The PPSH was obtained as a supplementary indicator for quantitative assessment.

In the cases of CPFE or pleural effusions, for example, mice confirmed biochemically and histologically as severe fibrosis in fact had unexpectedly lower or higher PPSH values. The histograms from those animals exhibited double peaks due to the air trapped into the damaged and dilated alveoli, or extraneous single polarized peak attributing to the existing of fluids at the lower part of the lung. Detailed comparisons between outlined CT histograms and histology were performed (data not shown). Moreover, a number of ways to viewing CT histograms were also utilized (*i.e.* ***Figure. A1***). As a result, mice identified with those outranged histograms will be excluded from fibrosis index analysis. Together, lung histogram profiling complemented and extended the qualitative radiological findings with a favorable sensitivity and specificity.

**Reference:**

1. Oliva IB, Cortopassi F, Rochester CL, Rubinowitz AN: **Combined pulmonary fibrosis and emphysema syndrome: a radiologic perspective.** *Monaldi Arch Chest Dis* 2011, **75:**220-234.

2. Jackson IL, Vujaskovic Z, Down JD: **Revisiting strain-related differences in radiation sensitivity of the mouse lung: recognizing and avoiding the confounding effects of pleural effusions.** *Radiat Res* 2010, **173:**10-20.

**Figure. A1**


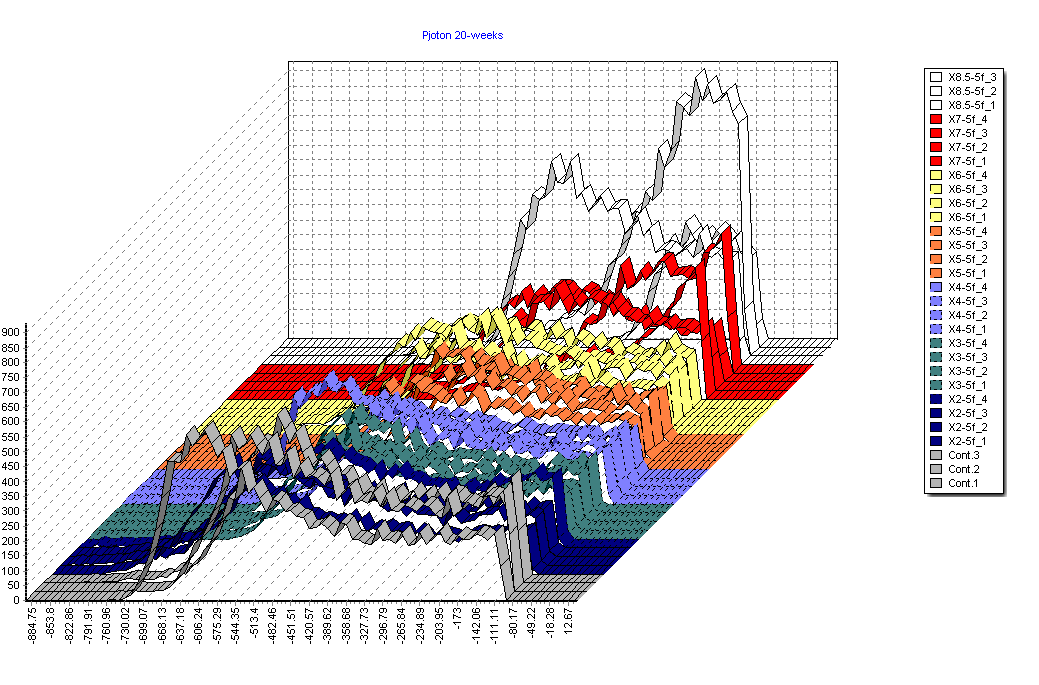


**IR Doses**

(each color represents one IR dose)
